# A Poor-Law Medical Officer and the Use of Brandy

**Published:** 1914-05-30

**Authors:** Thomas Dutton

**Affiliations:** 25 New Cavendish Street, Harley Street, W.


### THE EDITOR'S LETTER-BOX.

### A Poor-Law Medical Officer and the Use of Brandy.

*To the Editor of* The Hospital.

Sir,—It is refreshing to learn we have resident medical officers who will not allow interference with their professional duties towards the sick, and I congratulate Dr. W. T. G. Robinson on his firm stand with regard to stimulants in the workhouse under his control at Poole.

Guardians have not the slightest legal right to interfere with any treatment prescribed by their medical officer for the saving of life and the relief of pain in patients placed under their charge.

Brandy has always been looked upon as a drug by the profession, and a more useful diffusible stimulant is not to be found in the Pharmacopœia. I am certain I have saved a great many children's lives by the judicial use of it in proper doses and also old patients whose lives are ebbing away. It frequently revives patients long enough for us to prescribe other treatment or perform some operation.—I am, Sir, yours truly,

Thomas Dutton, M.D.

25 New Cavendish Street, Harley Street, W.
May 22, 1914.

